# MSC therapy in livestock models

**DOI:** 10.1093/tas/txac012

**Published:** 2022-01-27

**Authors:** Ellen M Harness, Nuradilla Binti Mohamad-Fauzi, James D Murray

**Affiliations:** 1 Department of Animal Science, University of California, Davis, One Shields Ave., Davis, CA 95616, USA; 2 Institute of Biological Sciences, Faculty of Science, Universiti Malaya, Kuala Lumpur 50603, Malaysia; 3 Institute of Ocean and Earth Sciences, Institute for Advanced Studies, Universiti Malaya, Kuala Lumpur 50603, Malaysia; 4 Department of Population Health and Reproduction, University of California, Davis, One Shields Ave., Davis, CA 95616, USA

**Keywords:** agriculture, dual-purpose model, in vivo model, livestock, mesenchymal stem/stromal cell, regenerative therapy

## Abstract

Mesenchymal stem cells (MSCs) have great value as therapeutic tools in a wide array of applications in regenerative medicine. The wide repertoire of cell functions regarding tissue regeneration, immunomodulation, and antimicrobial activity makes MSC-based therapy a strong candidate for treatment options in a variety of clinical conditions and should be studied to expand the current breadth of knowledge surrounding their physiological properties and therapeutic benefits. Livestock models are an appropriate resource for testing the efficacy of MSC therapies for their use in biomedical research and can be used to improve both human health and animal agriculture. Agricultural animal models such as pigs, cattle, sheep, and goats have grown in popularity for in vivo research relative to small animal models due to their overlapping similarities in structure and function that more closely mimic the human body. Cutaneous wound healing, bone regeneration, osteoarthritis, ischemic reperfusion injury, and mastitis recovery represent a few examples of the types of disease states that may be investigated in livestock using MSC-based therapy. Although the cost of agricultural animals is greater than small animal models, the information gained using livestock as a model holds great value for human applications, and in some cases, outcompetes the weight of information gained from rodent models. With emerging fields such as exosome-based therapy, proper in vivo models will be needed for testing efficacy and translational practice, i.e., livestock models should be strongly considered as candidates. The potential for capitalizing on areas that have crossover benefits for both agricultural economic gain and improved health of the animals while minimizing the gap between translational research and clinical practice are what make livestock great choices for experimental MSC models.

## INTRODUCTION

Mesenchymal stem cells (MSCs) have demonstrated great value as therapeutic tools in a wide array of biomedical applications in the field of regenerative medicine. Originally identified in 1970 as a cell population isolated from bone marrow that could form bone and fibroblast-like colonies (Friedenstein et al., 1970), years of study have elucidated the functions and therapeutic properties of MSCs. The International Society for Cellular Therapy (ISCT) characterizes MSCs as multipotent mesenchymal stem/stromal cells or medicinal signaling cells with varying cell surface marker expression across species and are capable of osteogenic, adipogenic, and chondrogenic differentiation in vitro (Dominici et al., 2006; Viswanathan et al., 2019), though differences in cell surface marker expression patterns are prevalent across species some similarities have been retained (Table 1). It is worth mentioning that limitations in availability of species-specific monoclonal antibodies hinder surface marker characterization of MSCs. Antibody reagent development in livestock should be prioritized to account for this, especially when MSCs are isolated from different tissue sources, i.e., bone marrow, adipose tissue, umbilical cord tissue, and placenta (Pittenger et al., 2019). Tissue sources have shown variability in proliferative capacity, viability, and level of activity between prenatal and adult origin (Hass et al., 2011). However, certain sources such placental and umbilical are non-invasive, and adipose is less invasive in collection procedures. Feasibility of isolation, culture conditions with limited and defined additives, and trophic activity allow for a multitude of disease states or injury to be treated via MSC-based therapy.

**Table 1.** Cell surface marker differences across species

Species	+Cell Surface Marker	-Cell surface Marker	Reference
human	**CD105**, **CD73**, **CD90,** CD44, CD29, CD166, CD117/c-Kit, CD71, (HLA)-ABC	**CD45**, CD34, CD14, CD11b, CD79alpha, CD19, HLA-DR, CD31	Dominici et al., 2006
			Rebelatto et al., 2008 Sotiropoulou et al., 2006
			Bourin et al., 2013
mouse	CD44, **CD90**, CD29, CD49e	**CD45**, CD11b, CD13, CD18, CD49d, CD19	Boregowda et al., 2016
			Eslaminejad and Nadri, 2009
			Meirelles and Nardi, 2003
pig	CD29, CD44, **CD90**, **CD73**, **CD105**, CD166	CD31, **CD45**, CD11b	Li et al., 2014
			Lapi et al., 2008
			Bosch et al., 2006
			Vacanti et al., 2005
			Ock et al., 2010
cattle	**CD105**, **CD90**, **CD73**	**CD45**	Gao et al., 2015
sheep	CD44, **CD90**, CD140a, **CD105**, CD166, CD29	**CD45**, CD14, CD31	Vivas et al., 2018
			McCarty et al., 2009
goat	**CD90**, **CD105**, **CD73,** CD44, CD29, CD166	**CD45**, CD34, CD14, CD79alpha, CD71	Mohamad-Fauzi et al., 2015
			Qiu et al., 2012
			Azari et al., 2011

Bolded markers indicate overlapping cell surface marker expression in MSCs.

**Figure 1.**
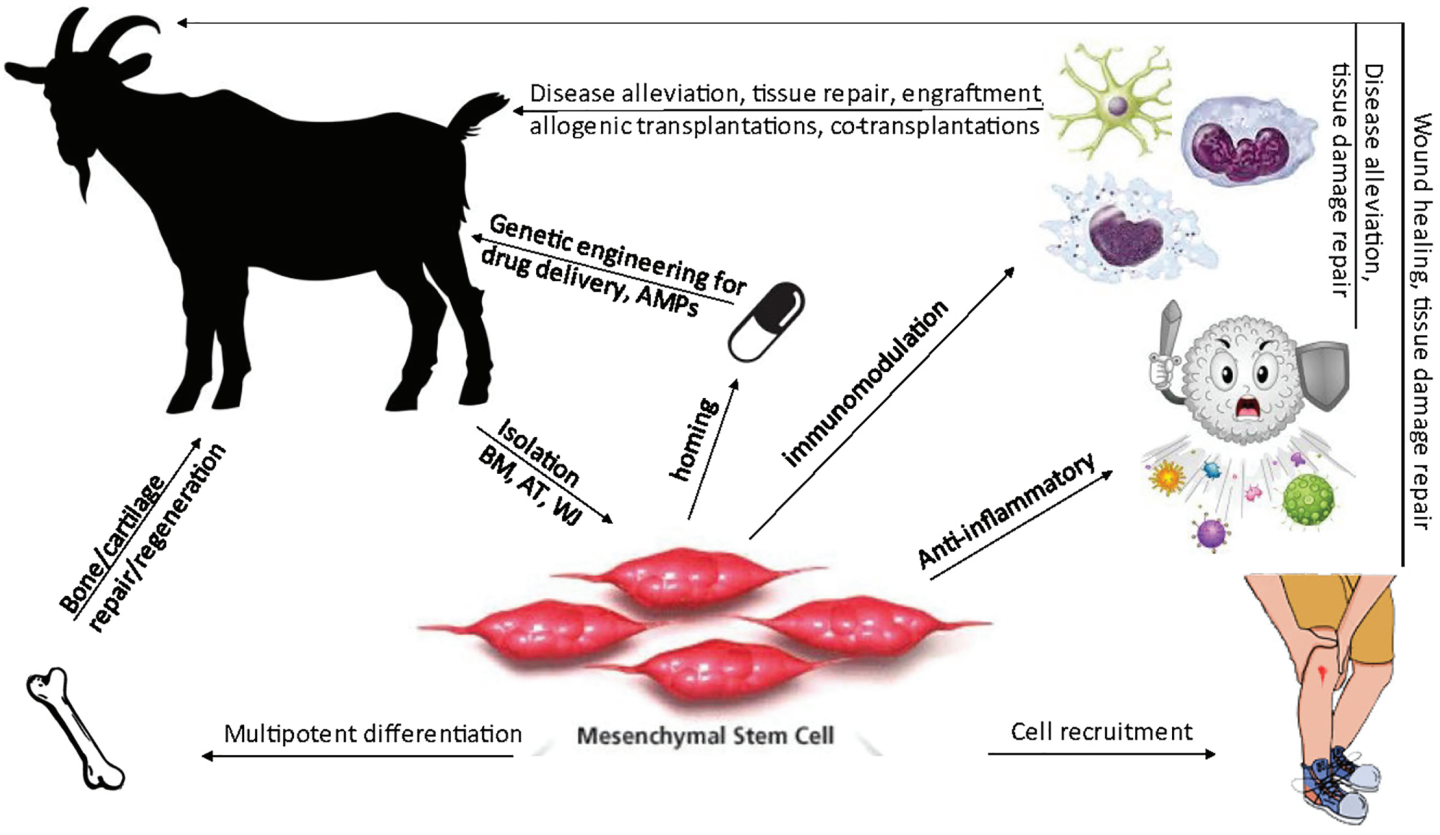
MSC-based applications that can be investigated using livestock models (Student’s adaptation from Millipore-Sigma website, The Scientist magazine, and Shutterstock).

MSCs are a highly studied adult stem cell for multilineage differentiation with potential in tissue regeneration applications. However, the therapeutic value of MSCs is primarily derived from secreted paracrine factors that influence surrounding tissue and cell populations to modulate the immune response, promote angiogenesis, facilitate reformation of the extracellular matrix, and stimulate progenitor cells to repair injured niches and restore tissue that was lost to injury or disease (for review see Wang et al., 2014 ; Moreira et al., 2017). Activated MSCs can create an anti-inflammatory environment in tissue to minimize further damage during regeneration and protect against infection by secreting antimicrobial peptides (AMPs) that inhibit bacterial growth and disrupt the integrity of bacterial cell membranes (Skalnikova et al., 2011; Harman et al., 2017). These characteristics vary slightly across species, but are primarily conserved regarding cell function, rendering MSCs an ideal cell type for regenerative therapies (Figure 1).

The incredible repertoire of functions makes MSC-based cell therapy a useful tool in biomedical applications. Expanding the current breadth of knowledge surrounding their physiological properties and therapeutic benefits remains necessary to fully understand paracrine signaling activity and cell to cell communication. Animal models provide a strong biological system in which to accomplish this objective. Livestock models represent an appropriate resource for testing the efficacy of MSC therapies, and their use in biomedical research can improve translational medicine and animal agriculture (Ireland et al., 2008; Cibelli et al., 2013; Roth and Tuggle, 2015). Small animal models are more readily available and typically have lower costs than agricultural animal models but differ from humans in numerous anatomical and physiological ways (Sullivan et al., 2001). Although cost of conducting an experiment is an important factor, physiological differences between the experimental in vivo system and the downstream application also must be considered for the information to translate. Agricultural animal models such as pigs, cattle, sheep, and goats have grown in popularity for in vivo research relative to small animal models due to their overlapping similarities in structure and function that more closely mimic the human body. Examples of in vivo studies for MSC therapy in livestock include targeting conditions such as osteoarthritis (Murphy et al., 2003), bone injury (Hill et al., 2019), diabetes (Peng et al., 2017), wound healing (Somal et al., 2017), cardiac repair (Whitworth and Banks, 2014), and spine repair (Gupta et al., 2007), which represent but a few examples of health conditions that could benefit from translational studies using livestock models (Table 2).

**Table 2.** Livestock species currently being used as in vivo models for experimental MSC therapies

Species	Condition/disease state	Reference
Pig	Cutaneous wound healing	Seaton et al., 2015
	Chronic ischemic	Bolli and Ghafghazi, 2015
	Cardiomyopathy	Mazhari et al., 2007
	Cardiac repair	Whitworth and Banks, 2014
	Osteoarthritis	Ando et al., 2007
	Cartilage repair	Lee et al., 2007
	Bone repair	Rubessa et al., 2017
Cattle	Bone injury	Hill et al., 2019
	Osteoarthritis	Wolfe, 2018
	Diabetes mellitus	Peng et al., 2017
Sheep	Spinal repair	Gupta et al., 2007
	Human MSC engraftment	Liechty et al., 2000; Shu et al., 2018
	Osteoarthritis	Al Faqeh et al., 2012
	Acute renal failure	Behr et al., 2007
Goat	Mastitis	Costa et al., 2019
	Osteoarthritis	Murphy et al., 2003; Nam et al., 2013
	Bronchopleural fistula	Petrella et al., 2014
	Microbial interactions	Foutouhi et al., unpublished data
	Wound healing	Somal et al., 2017; Pratheesh et al., 2017
	Germ cell generation	Zhang et al., 2019

Incorporating dual-purpose models, i.e., biomedical and farm animal research, for MSC therapies holds benefits for both clinical improvement for multiple conditions and supports agricultural institutions in practice (Ireland et al., 2008). Research involving agricultural animals requires special facilities and expertise in addition to the scientists who are familiar with the animals, and thus opens up the opportunity for funding to be granted to universities equipped with appropriate resources (Ireland et al., 2008). Funding for agricultural-based research maintains housing facilities, cost of food/water, access to veterinary care, research supply cost, and education opportunity for students, demonstrating the value and need for support financially in this field. The potential for capitalizing on areas that have crossover benefits for both agricultural economic gain and improved health of the animals while minimizing the gap between translational research and clinical practice is what make livestock great choices for experimental MSC models. In this review, we discuss MSC therapies for conditions and disease states being investigated using livestock models, and benefits gained by both the agricultural industry and translational clinical practice.

## ANIMAL MODELS FOR MSC THERAPY

### Pig

Pig MSCs derived in vitro from both adipose tissue and bone marrow show multilineage differentiation, differential gene expression, and cell surface marker expression (Table 1) (Kim et al. 2010; Monaco et al. 2010; Li et al., 2014). Pig models are very useful as they share similarities with humans in body size, anatomy, diet, and physiological/pathophysiological responses (Flisikowska et al., 2014). Pig MSCs from multiple tissue sources, i.e., bone marrow, adipose, and umbilical cord, have been used as models for MSC therapies for conditions such as cutaneous wound healing (Seaton et al., 2015), chronic ischemic cardiomyopathy (Bolli and Ghafghazi, 2015), cardiac repair (Whitworth and Banks, 2014), osteoarthritis (Ando et al., 2007), cartilage repair (Lee et al., 2007), and bone repair (Rubessa et al., 2017) to name a few.

Pig skin more closely mimics human skin composition than the skin of small rodents, heals through physiologically similar processes, and is an excellent model for the study of topical antimicrobial agents. This makes pigs an ideal animal model for human cutaneous wound healing over rodents who have fur, differentially structured dermal layers, and differing wound healing processes (Sullivan et al., 2001). Treatment of chronic nonhealing wounds, diabetic wounds, burns, and hypertrophic scars with MSCs have been modeled in pigs (Seaton et al., 2015). In pigs alone there are over 1500 peer reviewed publications to study wound healing and the pathophysiology of various wound types for translational human studies (Seaton et al., 2015).

Pig hearts more closely resemble human hearts than do the hearts of small rodents and therefore are a more appropriate model for translational research on cardiac repair. Unlike mouse hearts, human hearts do not beat 500 to 700 times/min, and repair of human myocardial infarctions requires replacing a few grams of tissue rather than a few milligrams, requiring large extrapolations for humans to be made from studies using mouse models (Bolli and Ghafghazi, 2015). Pig models of myocardial infarction and subsequent cardiac repair are much more appropriate given perspective. Pig models mimicking these disease states have demonstrated that MSC intervention stimulates endogenous repair by inducing the regeneration of stem cell niches in the heart (Mazhari et al., 2007).

MSC-based tissue-engineered constructs have been generated to facilitate repair of articular cartilage damage in pigs, and treatment with this therapy showed that the repaired tissue had similar mechanical properties to normal pig cartilage in static compression and friction tests (Ando et al., 2007). Alternative methods of treatment, especially those that minimize invasiveness, to improve the delivery of MSCs for therapy are important for optimizing translational practice, and large animal models such as the pig have provided the platform to test modes of MSC administration. Direct intra-articular injection of MSCs has been tested in pigs as a means of repairing cartilage defects and showed improved healing both histologically and morphologically (Lee et al., 2007).

Pig models for bone regeneration that incorporate MSCs lend themselves to improved techniques that surpass the limitations of bone grafting, i.e., donor site morbidity, rejection, and limited tissue regeneration (Rubessa et al., 2017). A pig model for bone injury resulting from a hole punched in the mandible showed distinct differences in tissue regeneration between MSC treatment and control groups where regeneration occurs in the presence of MSCs to a noteworthy extent (Wilson et al., 2012). MSCs injected at the injury site and MSCs injected intravenously showed similar regeneration, illustrating that MSCs injected intravenously were able to home to the injury site through the bloodstream. Wilson et al. (2012) also used GFP+ cells in this study, showing indisputable evidence of tissue growth from injected MSCs. The use of pigs as a model for MSC applications helps us to improve the efficacy and effectiveness for translational regenerative models and should be strongly considered for future investigations as an appropriate in vivo model for testing therapies.

### Cattle

Cattle MSCs as reviewed by Gao et al. 2015 are multilineage differentiation capable and express CD105, CD90, and CD73, while lacking expression of CD45. Cattle are large ruminants and less similar in size and anatomy to humans than other livestock models; however, conditions that affect both cattle and humans may prove useful for testing MSC therapies. For example, bone injury is a factor to consider for rate of recovery times in important stock, i.e., high-end breeders (Hill et al., 2019). Time is valuable regarding the cost of raising livestock, as is the genetic potential an individual can pass on to future generations for meat and milk production. Lost time has a substantial impact if important animals are subject to injury or disease that removes them from the breeding pool. MSC therapy can be implemented to improve the recovery time from bone injury and protect top tier breeders from losing time in passing on valuable genetics while providing a model to research bone injury in agricultural institutions (Hill et al., 2019;Bähr and Wolf, 2012). A second example is osteoarthritis, which affects both humans and livestock, and has been linked to infertility in bulls (Wolfe, 2018). MSC therapy for affected animals would improve quality of life, mobility, and protect high value genetics from being lost due to the disease state (Wolfe, 2018), while gaining additional knowledge applicable to translational medicine.

Conditions such as mastitis have direct impact on production potential in the dairy industry and provide an excellent model for alleviation of disease using MSC therapy for both tissue regenerative purposes and antimicrobial activity. Recovery time from mastitis not only influences a cow’s overall health, but contributes to overall production yield and collective economic gain. Genetically engineered epithelial cell lines that secrete AMPs, which are subsequently injected into affected quarters of the udder, have successfully been used to treat mastitis in cattle (Sharma et al., 2017), but MSCs have not been implemented as part of this cell therapy. MSCs have a long lifespan in culture and are feasibly altered using genetic engineering methods. Their natural homing ability to sites of inflammation and injury provides the opportunity to optimize the cell delivery method in a system that has already proven effective (Luo et al., 2013). Expanding MSC applications in this manner benefits livestock health and improves economic gain in the dairy industry.

Reducing recovery time from bone injury, osteoarthritis, mastitis, or any production related disease—all conditions for which MSCs have potential as treatment—is crucial for improved agricultural growth and profitability. The cattle industry has arguably the largest economic interest regarding animal agricultural production, strengthening the validation for choosing cattle as research models with the interest to increase efficiency and decrease losses in production yield while improving translational therapeutic approaches in MSC therapy.

### Sheep

Sheep MSCs as isolated and characterized by Vivas et al. (2018) are multilineage differentiation capable and expressive of currently recognized MSC cell surface markers. Sheep models for MSC therapy may be implemented for testing treatment and recovery for a number of conditions such as spinal repair (Gupta et al., 2007; Shu et al., 2018), bone graft substitutes (Liechty et al., 2000), osteoarthritis (Al Faqeh et al., 2012), and acute renal failure (Behr et al., 2007). Large animal models for bone injury, i.e., spinal, more closely mimic the size and healing rates of human bones than would small rodent models, which is an important factor to consider regarding systems for optimizing/improving surgical methods to be translated to humans. Bone graft substitutes have been successfully investigated in sheep models aimed at accelerating spinal fusion rates using osteoconductive graft material enriched with autologous MSCs rather than iliac crest autografts (Gupta et al., 2007). Sheep are more easily managed monetarily along with size and raising/experimental costs allowing for more investigative bone repair models for a ruminant. In this manner, sheep are translatable to both cattle and human investigation.

Efficacy of MSC engraftment in sheep models of acute renal failure modeled with experimental ischemia reperfusion injury, i.e., damage following blood flow returning to tissue in oxygen deprived state, has also been investigated to assess the outcome of time differential between injury and cell transplantation (Behr et al., 2007). MSCs injected into the renal artery showed renal engraftment in both tubules and glomeruli, expressed tubular epithelial cell markers and podocyte phenotype, and demonstrated a marked difference in level of engraftment based on time between injury and injection (Behr et al., 2007). MSC intervention in this medical condition can have a significant impact on reducing morbidity and mortality in patients exposed to prolonged ischemia, such as during surgery or myocardial infarction (Collard and Gelman, 2001). Inflammation appears to be a large contributor to the negative impacts of ischemia-reperfusion injury (Wu et al., 2018), which may be modulated by MSC-secreted factors. Ischemia-reperfusion injury is associated with serious clinical manifestations and is worth further investigating in livestock how MSCs may be used as treatment options for these cases.

Human MSC engraftment has been conducted in fetal sheep models to study the multipotential capacity after transplantation and unique immunologic characteristics that have important implications for clinical utility in cellular and gene therapies using MSCs (Liechty et al., 2000). MSC therapy for osteoarthritis has been examined using sheep models, where chondrogenically induced MSCs were injected into the knee joint of an osteoarthritic animal and showed a reduction in progression of the disease state (Al Faqeh et al., 2012). Sheep are rather versatile in the conditions that can be studied for human translation and should not be undervalued as a choice for an in vivo model for MSC therapy.

### Goat

Goat MSCs as characterized by Mohamad-Fauzi et al. 2015 are capable of tri-lineage differentiation, express cell surface markers CD90, CD105, and CD73, and lack expression of the hematopoetic marker CD45. Similar to sheep, their anatomical  and physiological similarity to humans has made goats a frequently used large animal model for MSC therapy to treat a variety of conditions such as mastitis recovery (Costa et al., 2019), osteoarthritis (Murphy et al., 2003), and lung injury (Petrella et al., 2014).

Mastitis directly impacts the dairy industry by reducing the number of animals available for milk production and may result in irreversible changes in the mammary gland tissue that influences the animal’s ability to produce at the same capacity in the future (Costa et al., 2019). Studies using MSC therapy for mastitis treatment in goats by direct injection of MSCs into affected udders demonstrated improved functionality of the mammary gland and showed alterations of tissue organization that mimic alveoli development, the presence of fluid, and restored standard milk fat composition (Costa et al., 2019). This type of MSC therapy has crossover benefits not only for dairy goats and dairy cattle recovering from mastitis, but can directly impact an animal’s future ability to produce milk at a comparable quantity and quality prior to the disease state. On a large industrial scale for production, this type of treatment could provide significant economic gain by shortening an animal’s recovery time and protecting their potential volume of milk produced over time and thus impacting human food security.

Overlapping osteochondoral conditions between humans and goats, such as osteoarthritis or defects from traumatic injury, are feasibly researched due to similarities in structure of the knee joint (Murphy et al., 2003; Proffen et al., 2012). MSC treatment of goats having induced-osteoarthritic joints showed regeneration of the medial meniscus and detection of implanted cells in newly formed tissue (Murphy et al., 2003; Nam et al., 2013). Disease states such as bronchopleural fistula, or persistent communication between the bronchial tree and pleural space that can arise as a complication from surgery, trauma, or injury to the lungs have been investigated using goats as the in vivo system due to an anatomically similar pleural cavity and mediastinum (Chhangani, 2008; Petrella et al., 2014). Studying tissue regeneration and rate of recovery time in this system translates well to MSC therapy in human patients and may have profound impacts on recovery rate from surgical or secondary complications that cause bronchopleural fistula (Petrella et al., 2014). Experimental outcomes such as this hold great value for safety and efficacy testing in developing methods for the administration of cell therapy in human clinical trials.

Size, structure, and function of the in vivo species used as a model remain important aspects to study disease and tissue damage for translational information to be gained (Bascunan et al., 2019). Goats also may be a more feasible in vivo option than larger animals such as cattle due to their smaller size and lower overall cost of maintenance. MSC therapy using goats as experimental models has numerous benefits for both the agricultural industry and human clinical treatments, rendering them as an excellent choice for an in vivo model.

## DISCUSSION

There is a wide array of research areas underway for MSC therapies, especially with new discoveries being made surrounding cell therapy, conditioned media applications, and MSC-derived exosomes in practice, demonstrating the need for appropriate biological systems to test the safety and efficiency of treatment methods. The models described in this review demonstrate safe and advantageous systems in which to pursue further investigation into alternative methods of therapy for MSC-based regenerative medicine. It is imperative to recognize the value of large animal models in biomedical research to benefit both animal and human health, as well as to impact the quality of life, alleviation of disease, production, and economic gain in the agricultural industry (Ireland et al., 2008). Arnold Caplan refers to MSCs as medicinal signaling cells due to their migratory and trophic activity in situ (Caplan, 2017). A substantial part of the repertoire of bioactive factors provided by MSCs comes in the form of exosomes, tiny multivesicular bodies that contain therapeutic factors packaged and released from the MSC to surrounding cells for downstream messaging or biochemical function (Toh et al., 2018). Exosome-based paracrine signaling and/or cell to cell communication are being investigated as new insights into RNA and protein-based mechanisms of action are uncovered. Advantages of exosome-based therapy revolve around influencing surrounding cell populations like that of conditioned media or cell-based therapy with the difference being that exosomes are packages of therapeutic substrate that can be isolated. Concentrated secreted factors with the appropriate delivery system could in theory increase the rate of recovery for patients. Exosomes have been demonstrated to have therapeutic efficacy against a myriad of disease including liver fibrosis (Li et al., 2013), ischemic reperfusion (Lai et al., 2010), cartilage regeneration (Toh et al., 2016), and cutaneous healing (Wu et al., 2018) as examples. Relative time between onset of injury to initiation of treatment is important in wound healing and tissue regeneration. Exosomes functionally could bypass the time needed for migration and homing in cell-based therapy as well as the time to respond to surrounding damaged tissue populations and initiate downstream signaling cascades (Pittenger et al., 2019). This could influence the efficacy of treatment and regeneration time in patients, and is an important factor to consider in the future of MSC investigation for regenerative therapy.

Livestock species such as goats, sheep, pigs, and cattle should be strongly considered as an in vivo experimental system for future investigation of MSC applications in both human translational practice and agricultural production settings. General areas of human translational research that could benefit from using large animal models that were not covered in this review include aging, biomechanics, cardiovascular disorders, cancer biology, comparative physiology, diabetes (types I and II), transmissible diseases, liver disorders, immunology, microbial ecology, neurobiology, nutrition, obesity, ophthalmology, pregnancy, radiation biology, renal biology, therapeutics, toxicology, and reproduction (Ireland et al., 2008). Some of these areas have been investigated using MSC-based livestock models and have shown promising potential for the application of cell therapies (see Table 2). Other areas could benefit by exploring MSC therapy for disease alleviation and improved recovery times following cell or secreted cell product administration. Recent advances and therapies being explored with MSCs include immune invasion related to cancer and allogenic therapy (Miranda-Rodríguez et al., 2016; Poggi et al., 2014), and would greatly benefit in understanding the depth of efficacy by incorporating livestock models for in vivo experimentation. Pre-human clinical trials are necessary for the safety of patients and the value of information gained from larger animal models holds more biological proof of principle regarding accuracy of the treatment method and expected outcome for patients.

MSC models using livestock would benefit the translational aspect to humans but also serve the dual-purpose of potentially benefiting farm animal health as well. By modeling disease in livestock, or applying the therapy to a naturally occurring clinical condition, researchers may improve methods for treating animals and shorten recovery time for important individuals, i.e., high-value breeding stock while optimizing methods of disease treatment that can cross over into human practice. Retention of production animals, regarding both meat and milk, is an incredibly important aspect of the agricultural industry due to the time, cost, and labor of raising those animals to market weight, or longevity of those animals in breeding herds. Knowledge gaps to address related to this field of research include antibody reagent availability for livestock for appropriate cell characterization, tissue source differences from which MSCs are derived, and species-specific differences in MSC function. Cell surface marker characterization (Table 1) remains an important aspect in identifying populations of MSCs, and availability of monoclonal antibodies with species specific surface markers for testing remains a challenge in livestock. Differences in MSCs derived from bone marrow, adipose tissue, Wharton’s jelly, umbilical cord, placenta, etc., also may play an important role in MSC application, and should be further elucidated in livestock species to better understand the role between differences in tissue source origin, cell function, and downstream therapy, i.e., how tissue source impacts cell function as well as relative age of the donor. Future outlooks related to addressing these gaps would include expanding the understanding of in vitro–based MSC therapies, and an expanded repertoire of in vivo models that have more biologically comparable data to human medical conditions. Utilizing livestock as research models benefits herd health, assists to improve economic gain for producers, and provides a system for testing MSC treatments. Dual-purpose models, therefore, should strongly be considered for future investigation in MSC-based therapies.

## CONCLUSION

Using livestock models as research tools for MSC-based therapies holds benefits for both human biomedical applications and production outcomes in the agricultural industry. The information gained using livestock as a model holds great value for human applications, and in some cases, outcompetes the weight of information gained from small rodent models. Although the cost of agricultural animals is greater than small animal models, the efficacy of therapies may be more translational to clinical practice and could thus bring funding to those universities with the facilities and expertise to carry out the research. Providing funding to agricultural universities is critical to gain that valuable information and provide cost for research materials, housing, maintenance, veterinary care, and education opportunities for students to participate in livestock-based MSC research projects. Research surrounding livestock and MSC regeneration has demonstrated value in cell and conditioned media applications, and should incorporate more techniques such as exosome-based therapy for downstream investigation. Many aspects of MSC-based therapy have promising potential and should be investigated with the appropriate in vivo models, such as livestock, for an expanded understanding of regenerative applications in translational medicine.
